# Exploration of the Shared Molecular Mechanisms between COVID-19 and Neurodegenerative Diseases through Bioinformatic Analysis

**DOI:** 10.3390/ijms24054839

**Published:** 2023-03-02

**Authors:** Yingchao Shi, Wenhao Liu, Yang Yang, Yali Ci, Lei Shi

**Affiliations:** 1 State Key Laboratory of Medical Molecular Biology, Institute of Basic Medical Sciences, Chinese Academy of Medical Sciences and School of Basic Medicine, Peking Union Medical College, Beijing 100005, China; 2 Department of Biochemistry and Molecular Biology, Institute of Basic Medical Sciences, Chinese Academy of Medical Sciences and School of Basic Medicine, Peking Union Medical College, Beijing 100005, China; 3 Department of Neurology, Peking Union Medical College Hospital, Chinese Academy of Medical Sciences and Peking Union Medical College, Beijing 100005, China

**Keywords:** COVID-19, Alzheimer’s disease, Parkinson’s disease, bioinformatics, differentially expressed genes, gene ontology, protein–protein interaction, hub genes, drugs

## Abstract

The COVID-19 pandemic has caused millions of deaths and remains a major public health burden worldwide. Previous studies found that a large number of COVID-19 patients and survivors developed neurological symptoms and might be at high risk of neurodegenerative diseases, such as Alzheimer’s disease (AD) and Parkinson’s disease (PD). We aimed to explore the shared pathways between COVID-19, AD, and PD by using bioinformatic analysis to reveal potential mechanisms, which may explain the neurological symptoms and degeneration of brain that occur in COVID-19 patients, and to provide early intervention. In this study, gene expression datasets of the frontal cortex were employed to detect common differentially expressed genes (DEGs) of COVID-19, AD, and PD. A total of 52 common DEGs were then examined using functional annotation, protein–protein interaction (PPI) construction, candidate drug identification, and regulatory network analysis. We found that the involvement of the synaptic vesicle cycle and down-regulation of synapses were shared by these three diseases, suggesting that synaptic dysfunction might contribute to the onset and progress of neurodegenerative diseases caused by COVID-19. Five hub genes and one key module were obtained from the PPI network. Moreover, 5 drugs and 42 transcription factors (TFs) were also identified on the datasets. In conclusion, the results of our study provide new insights and directions for follow-up studies of the relationship between COVID-19 and neurodegenerative diseases. The hub genes and potential drugs we identified may provide promising treatment strategies to prevent COVID-19 patients from developing these disorders.

## 1. Introduction

With the liberalization of epidemic prevention and control in various countries, more than 600 million people worldwide have been diagnosed with coronavirus disease 2019 (COVID-19), which is caused by the novel Severe Acute Respiratory Syndrome Coronavirus-2 (SARS-CoV-2). It is well known that SARS-CoV-2 mainly attacks the human respiratory system and causes typical symptoms, including fever, sore throat, cough, shortness of breath, and fatigue. Moreover, current evidence supports that SARS-CoV-2 is capable of targeting and invading the central nervous system (CNS) [1]. Neurological symptoms have been observed during and after the acute COVID-19 phase, including both CNS symptoms and vegetative/peripheral manifestations [2]. In particular, recent studies have suggested that COVID-19 may trigger clinical manifestations of neurodegenerative disorders, such as cognitive decline [3], dementia [4], and parkinsonism [5], bringing the potential role of COVID-19 in the future development of neurodegenerative diseases into the spotlight. Some studies reported increased risk of these disorders among COVID-19 positive patients [6,7]. The changes in COVID-19 patients’ brain structure also reinforced this hypothesis [8]. In addition, COVID-19-induced impairment of the frontal cortex, a critical area for cognitive function, was described in complementary studies that combined neuro-imaging and cognitive screening [9].

Neurodegenerative diseases are characterized by progressive dysfunction and loss of neurons [10], and they can affect an individual’s movement, speech, memory, cognition, intelligence, and much more [11,12]. These diseases include Parkinson’s disease (PD), Alzheimer’s disease (AD), Huntington’s disease (HD), multiple sclerosis (MS), amyotrophic lateral sclerosis (ALS), epilepsy, and others [13]. AD and PD are the two most common human neurodegenerative diseases, and AD is the leading cause of dementia. Although PD is traditionally considered a movement disorder, dementia is becoming more widely accepted as part of the clinical spectrum of PD [14]. A previous study found that mild cognitive impairment (MCI) was one of the most common non-motor symptoms of early-stage PD patients, and dementia was presented in 83% of 20-year PD survivors [15]. There is growing evidence suggesting an association between AD and PD at the molecular level, such as failure in redox homeostasis, improperly folded modified proteins, and neuroinflammation [16]. The damage to the frontal cortex has been implicated in both AD and PD [17,18]. Clinical studies have reported that patients with a previous neurodegenerative disease have an increased risk for COVID-19, as well as COVID-19-related hospitalization and mortality [19,20,21]. Progress in deciphering the common pathogenesis of COVID-19, AD, and PD is conducive to developing effective strategies to treat the neurological symptoms of infected individuals and to prevent these patients from developing neurodegenerative diseases.

**Figure 1:**
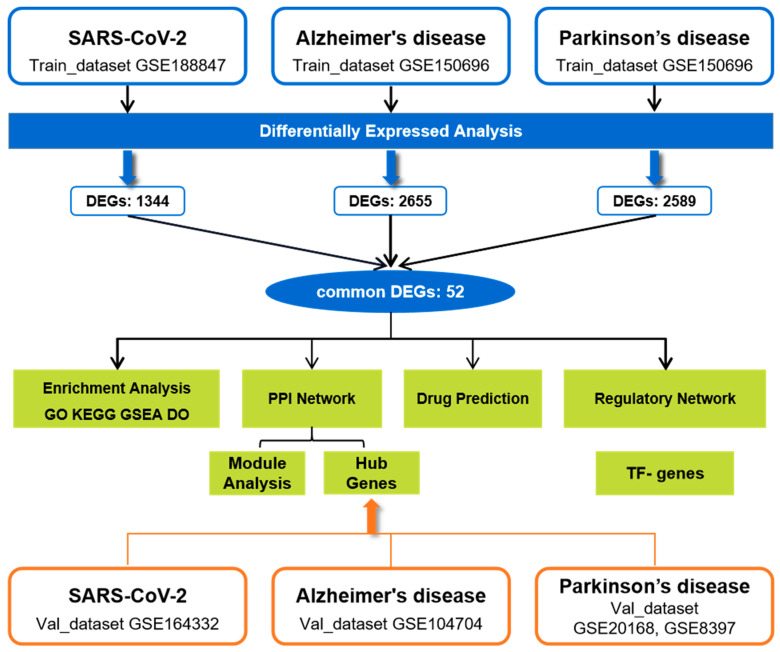
The workflow of this study.

To explore the molecular mechanisms of COVID-19-related neurodegenerative symptoms, we estimated transcriptome alterations in the frontal cortex of patients with COVID-19, AD, and PD using two datasets. Further analyses, including Gene Ontology and pathway enrichment, protein–protein interaction (PPI) and key module extraction, identification of hub genes and potential drugs, and transcription factor (TF) regulatory network construction, were performed based on the common DEGs among COVID-19, AD, and PD. The sequential workflow of our research is presented in Figure 1.

## 2. Results

### 2.1. Identification of DEGs and Common DEGs among COVID-19, AD, and PD

**Figure 2:**
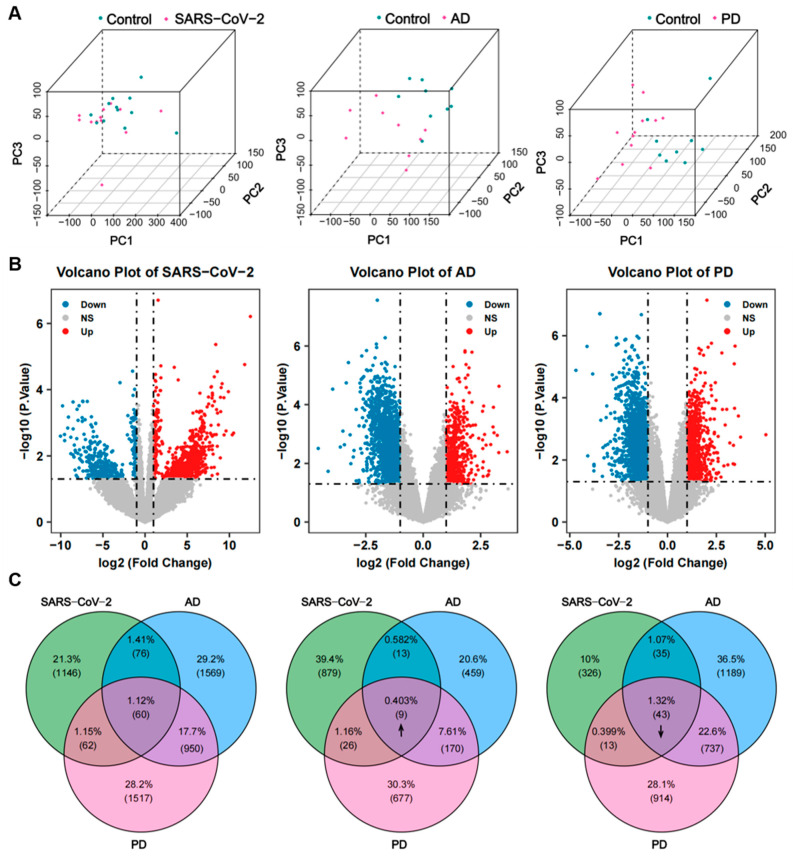
Identification of common DEGs shared by COVID-19, AD, and PD. (**A**) PCA plots of the COVID-19, AD, and PD expression datasets used in this study after removing batch effects and normalization. (**B**) Volcano plots of the three datasets. Up-regulated genes are marked in red, and down-regulated genes are marked in blue. (**C**) Venn diagram (**left**) reveals that 60 common DEGs are shared among COVID-19, AD, and PD: 9 genes are consistently up-regulated (**middle**, up arrow) and 43 genes are consistently down-regulated (**right**, down arrow) in the 3 datasets.

To determine shared genetic interrelations among COVID-19, AD, and PD, we initially accessed the transcriptomic data from the frontal cortex of each disease in the GEO database. Before the procedure of differential analysis, we performed normalization and removal of batch effects to standardize the expression matrices, and the results of the processing are shown in a density plot (Supplementary Figure S1A,B). After standardization, the normality test (Supplementary Figure S1C) and the PCA plot of each dataset (Figure 2A) indicate that the source of samples is reliable. Next, differential analysis of gene expression was performed by controlling age and sex, which was significantly different between the patients and the healthy controls (Table 1, Table 2 and Table 3). Finally, 1344 genes were identified as DEGs for COVID-19, including 927 up-regulated and 417 down-regulated genes. In the same way, 2655 DEGs (651 up-regulated and 2004 down-regulated) in the AD dataset and 2589 DEGs (882 up-regulated and 1707 down-regulated) in the PD dataset were obtained. The results are shown in the volcano plots (Figure 2B). Using a cross-comparative analysis, we identified 52 common DEGs, including 9 common up-regulated genes and 43 common down-regulated genes, after excluding genes with opposite expression trends among COVID-19, AD, and PD (Figure 2C). This common gene set was submitted to further experiments.

### 2.2. Functional Annotation and Pathway Enrichment Analysis of Common DEGs

The connectivity of common DEGs may indicate crucial information about similar biological roles. To further understand the underlying common biological characteristics among COVID-19, AD, and PD, we implemented four canonical and widely used databases to analyze the common DEGs, including GO, KEGG pathway, Reactome, and GSEA.

**Figure 3:**
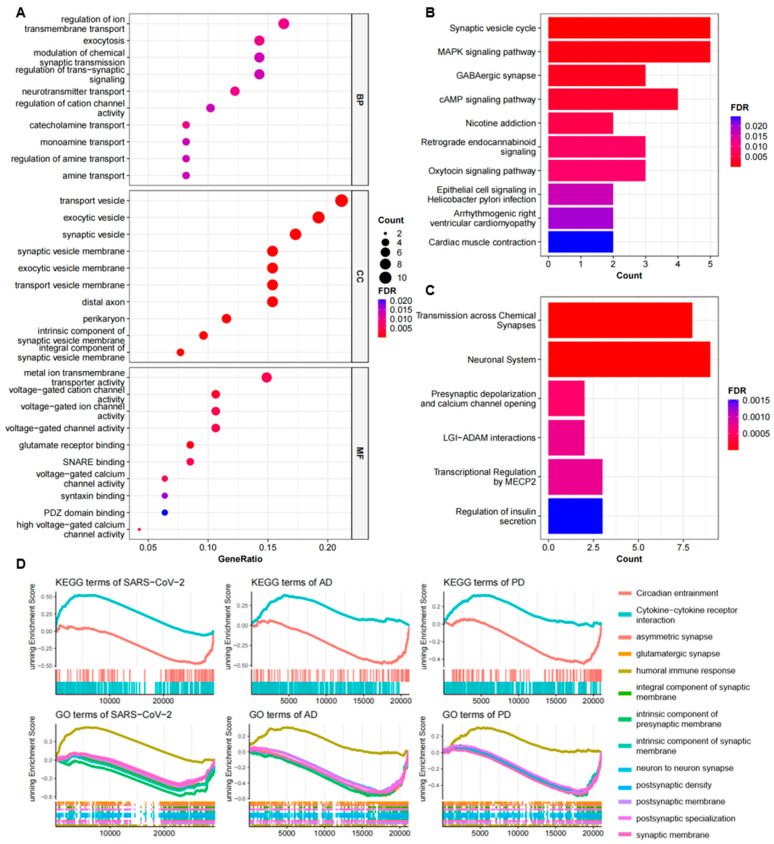
Functional annotation of common DEGs among COVID-19, AD, and PD. (**A**) GO analysis of shared DEGs and the top 10 terms of each category, including biological process, molecular function, and cellular component, are shown in the dot graph. (**B**,**C**) The pathway enrichment analysis results of the KEGG (**B**) and Reactome (**C**) databases. The top 10 pathways are exhibited in the bar plots. Count represents the number of DEGs enriched by the term. (**D**) The GSEA of the three datasets. The enriched common KEGG and GO terms shared by COVID-19, AD, and PD are shown here.

Typically, GO enrichment analysis is performed to identify the most important molecular features associated with genes, which can be categorized into three subsections, including biological process (BP), molecular function (MF), and cellular component (CC), for the annotation of gene products. For the biological process, the top GO terms that we enriched were associated with synaptic signaling and its regulation, such as neurotransmitter transport, modulation of chemical synaptic transmission, catecholamine transport, and regulation of trans-synaptic signaling. According to the cellular component, synaptic vesicle, transport vesicle, exocytic vesicle, and distal axon were the top terms. In the molecular function, voltage-gated ion channel activity was the main enriched GO term. The top 10 GO terms of each subsection are illustrated in a dot graph (Figure 3A) and summarized in Table 4.

For the pathway enrichment analysis, the KEGG analysis revealed that these common DEGs were significantly enriched in the synaptic vesicle cycle pathway, GABAergic synapse, MAPK signaling pathway, cAMP signaling pathway, and nicotine addiction (Figure 3B). The Reactome analysis showed that these genes were most related to transmission across chemical synapses, neuronal system, presynaptic depolarization, calcium channel opening, LGI-ADAM interactions, transcriptional regulation by MECP2, and regulation of insulin secretion (Figure 3C). Further independent analysis for the common up-regulated and down-regulated DEGs revealed that the GO terms and pathways mentioned above were mostly down-regulated, suggesting that a dysfunction of the synaptic vesicle cycle might be the common pathogenesis of COVID-19, AD, and PD. More information for the pathway enrichment results is presented in Table 5.

In addition, we used the GSEA to analyze common up-regulated and down-regulated GO terms and KEGG pathways in the COVID-19, AD, and PD expression datasets. The results demonstrated that cytokine–cytokine receptor interaction and humoral immune response were up-regulated. The down-regulated terms were mainly linked to synapses, synaptic membrane, and synaptic properties (Figure 3D). Based on these findings, we supposed that SARS-CoV-2 infection might cause a general down-regulation of genes associated with the synaptic vesicle cycle and synaptic signal transmission in the patients’ frontal cortex.

### 2.3. Gene–Disease Analysis of Common DEGs

**Figure 4:**
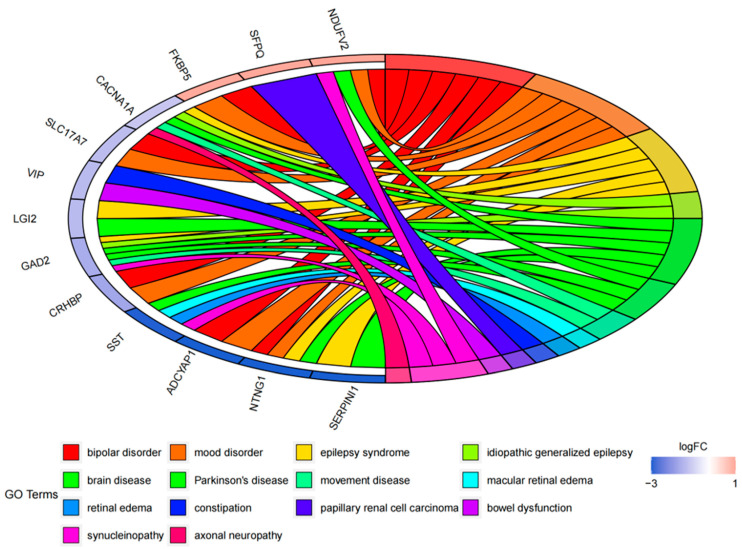
DO enrichment analysis. The chord diagram shows the correlation between diseases and common DEGs, with different colors corresponding to different DO terms.

The DO (Disease Ontology) enrichment analysis was conducted to identify the diseases associated with the common DEGs, thereby providing novel perspectives on our intended diseases. Through the DO analysis, we found that the common DEGs were mainly related to a loss of cognitive function or mental diseases, such as different types of epilepsy, dementia, and autism disorder, supporting that these common DEGs might be involved in the neurological symptoms of COVID-19, AD, and PD (Figure 4).

### 2.4. PPI Network Construction and Key Module Analysis

**Figure 5:**
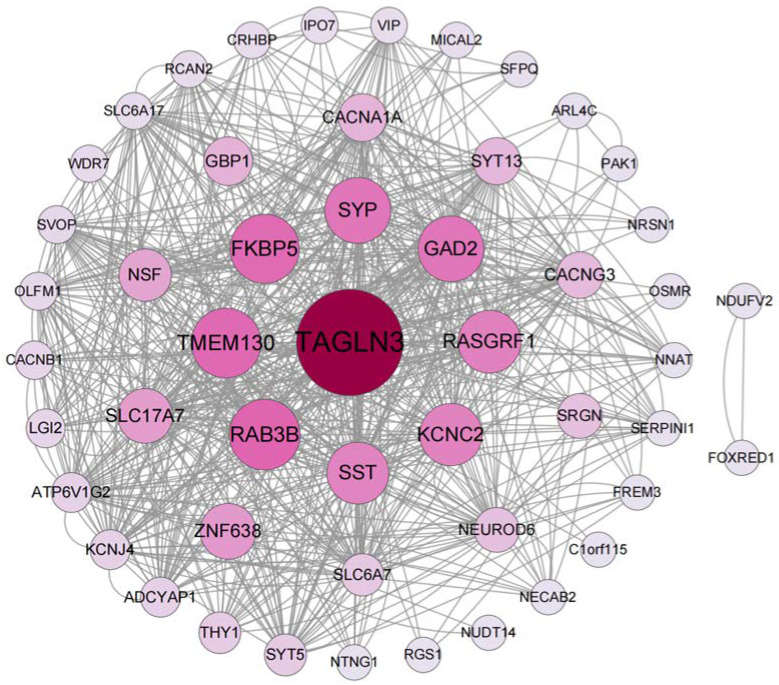
The PPI network of the identified common DEGs among COVID-19, AD, and PD. The network, including 52 nodes and 320 edges, is generated using String and visualized in Cytoscape. The circle nodes represent the DEGs, and the edges represent the interactions between the nodes; the size and color depth of the nodes are based on the Betweenness Centrality (BC) values.

**Figure 6:**
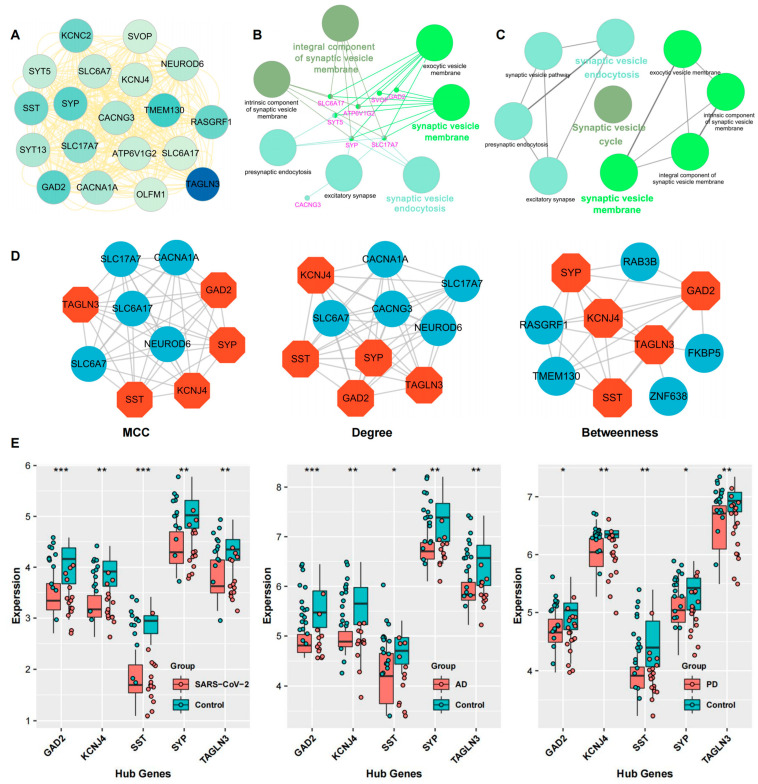
Detection of hub genes and key module from the PPI network. (**A**) The critical function module was extracted from the PPI network using the MCODE plugin in Cytoscape. (**B**,**C**) The GO annotation (**B**) and pathway enrichment analysis (**C**) of the key module. (**D**) The top 10 genes ranked by MCC, Degree, and BC algorithm. The common five genes, colored in orange, are determined as the hub genes. (**E**) The verification of the hub genes through the COVID-19, AD, and PD Val_datasets, respectively. *** *p* < 0.001, ** *p* < 0.01, * *p* < 0.05.

PPI networks have been used to discover novel protein functions, as well as identify functional modules and conserved interaction patterns [22]. Thus, the construction of a PPI network is regarded as the crucial step of cellular biology study and works as a precondition for system biology [23]. Here, the PPI network of the common DEGs is depicted in Figure 5, containing 52 nodes and 320 edges. Based on the PPI network, two closely connected modules were obtained through the MCODE plugin, and module 1 is shown in Figure 6A, which has the highest score (18.222) with 19 nodes. The GO and KEGG pathway analyses of module 1 were performed using ClueGO. The results of the GO analysis indicated that it was primarily related to synaptic vesicles (Figure 6B), and the KEGG pathway analysis also showed that module 1 was significantly correlated with the synaptic vesicle cycle (Figure 6C).

### 2.5. Identification of Hub Genes

According to the PPI network, we intended to explore the hub genes that play indispensable roles in the shared biological mechanisms of COVID-19, AD, and PD. Based on three widely used methods, MCC, Degree, and Betweenness Centrality, we listed each algorithm’s top 10 hub genes (Figure 6D). After taking the intersection of these genes, we identified five common genes as the hub genes, including *TAGLN3*, *GAD2, SST*, *SYP*, and *KCNJ4*.

### 2.6. Identification of Potential Therapeutic Drugs

**Figure 7:**
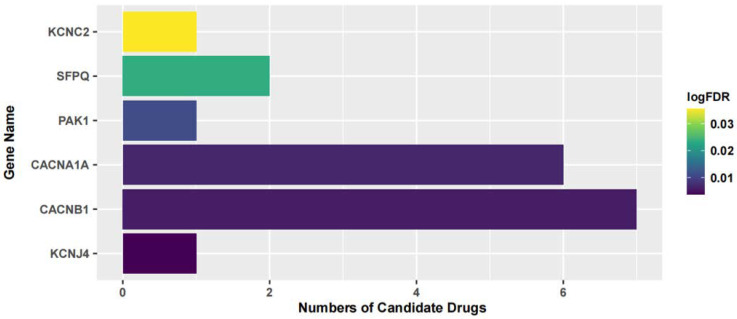
The relationship between the candidate drugs and their drug targets.

Furthermore, we searched the drug targets of the common DEGs to identify potential therapeutic targets. Here, we identified 6 drug targets and 18 related drugs based on DrugBank (Figure 7). Among them, five drugs, including Ibutilide, Azelnidipine, Dotarizine, Copper, and Artenimol, were considered to have potential therapeutic effects. The detailed information of these drugs and their targets is summarized in Table 6. Ibutilide is a class III antiarrhythmic agent used to correct atrial fibrillation and atrial flutter [24], and it can be considered as an alternative to cardioversion. Azelnidipine is a dihydropyridine calcium channel blocker [25]. It has a gradual onset of action and produces a long-lasting decrease in blood pressure, with only a small increase in heart rate. It is currently being studied for post-ischemic stroke management [26]. Dotarizine is a calcium antagonist used to treat and prevent migraines [27]. Copper is an essential element in the body and is incorporated into many oxidase enzymes as a cofactor [28]. The precise mechanisms of the effects of copper deficiency are vague due to the wide range of enzymes which use its ion as a cofactor. Artenimol is an artemisinin derivative and an antimalarial agent used in the treatment of uncomplicated *Plasmodium falciparum* infections [29].

### 2.7. Identification of Regulatory Transcript Factors

**Figure 8:**
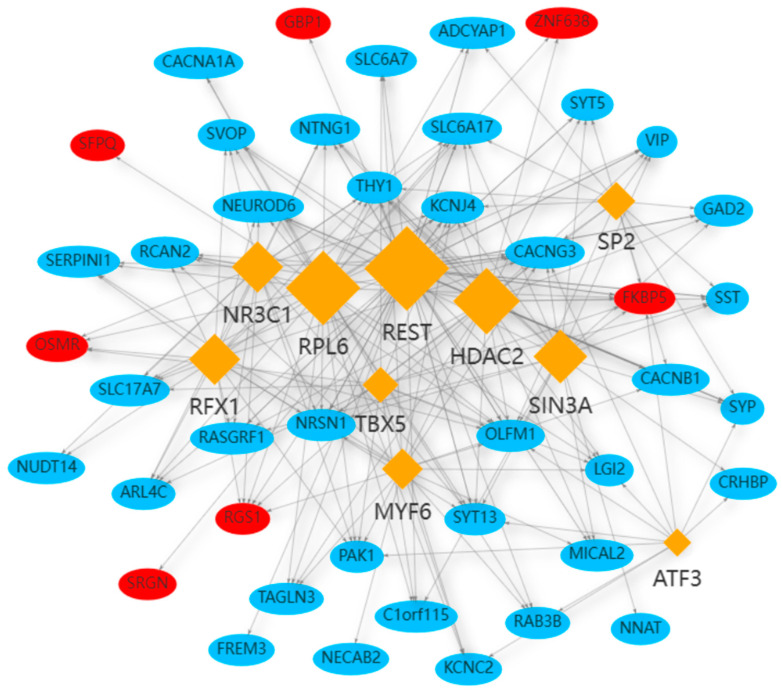
The network of TF–gene interaction. The blue color nodes represent the common genes, and the rhombus nodes represent the enriched TFs. The network consists of 10 TFs and 42 DEGs in total. The up-regulated DEGs are labeled in red, and the down-regulated DEGs are labeled in blue.

The mapping and characterization of TFs regulating the expression of common DEGs can provide insights into the comprehensive biological processes [30]. In this study, 10 possible TFs, including SP2, SIN3A, REST, ATF3, MYF6, TBX5, RFX1, RPL6, NR3C1, and HDAC2, were discovered to be related to 42 common DEGs (Figure 8). Among these DEGs, the five hub genes were all involved.

## 3. Discussion

In this study, we used three datasets of COVID-19, AD, and PD patients’ frontal cortex from the GEO database to discover the underlying mechanisms and potential therapeutic strategies for neurodegenerative disorders caused by SARS-CoV-2 infection. Through the intersectional analysis, we identified 52 common DEGs, and most of them were down-regulated, indicating that COVID-19, AD, and PD might cause a suppression of common cellular functions in patients’ frontal cortex.

We next performed a pathway-based analysis to identify shared biological pathways of COVID-19, AD, and PD. The pathway analysis revealed that these common DEGs were significantly enriched in the synaptic vesicle cycle pathway. Synaptic vesicles undergo a complex trafficking cycle, which could be divided into sequential steps: the formation of synaptic vesicles; the docking of synaptic vesicles in the active zone of the presynaptic membrane; the priming of synaptic vesicles; the fusion of synaptic vesicles with the presynaptic membrane; the release of neurotransmitters by exocytosis; and the endocytosis of vesicles [31]. An independent GO analysis of the common up- and down-regulated DEGs showed that the top terms, which were mainly associated with the synaptic vesicle cycle, were all down-regulated (Supplementary Figure S2A,B). Furthermore, the GSEA of the three datasets also demonstrated that synapses, components of synapses, and synaptic function were down-regulated in these three diseases. Our results indicated that the loss and damage of synapses and synaptic dysfunction might be the cause of neurodegenerative disease-related symptoms in COVID-19 patients or survivors.

Consistent with our results, some studies have shown that SARS-CoV-2 infection may cause synaptic disorder based on high-throughput sequencing and systematic bioinformatic analyses. Andrew et al. found that the synaptic signaling of upper-layer excitatory neurons, which are linked to cognitive function, is preferentially affected in COVID-19 patients through profiling large single-nucleus transcriptomes from the frontal cortex and choroid plexus samples across control individuals and patients with COVID-19 [32]. Cheng et al. also identified that SARS-CoV-2-infected neurons undergo degeneration, including shortened neurite length and reduced synapses [33].

AD may be primarily a disorder of synaptic failure. Synapse loss and synaptic dysfunction are the best-known pathological correlates of cognitive deficits found in AD patients [34,35]. Recently, studies have shown that synaptic pathology occurs in the early stage of AD progression, mainly manifested by a loss of synaptic proteins [36]. It has been reported that synaptophysin, a presynaptic vesicle protein, is decreased by around 25% in MCI patients, and this change occurs before Aβ plaque formation [37]. The dysfunction of synapses in the frontal cortex is considered a marker of AD progress and a very promising therapeutic target. Moreover, researchers have started to develop synaptic therapies to restore and prevent synaptic dysfunction in AD. These treatment strategies aim to avoid synaptic loss, strengthen synaptic connections, and improve synaptic signal transmission function. Moreover, recent studies have shown that a disorder of synaptic vesicle trafficking plays a vital role in the pathogenesis of PD [38]. Among the reported PD-related genes, *alpha-synuclein* (αS) [39,40], *LRRK2* [41,42], *Parkin* [43,44], *PINK1* [45,46], and *DJ-1* [47,48] have also been found to regulate the release of neurotransmitters from presynaptic vesicles and the circulation of synaptic vesicles. Obviously, synaptic dysfunction is closely related to the progression of neurodegenerative diseases.

Collectively, previous studies have shown that the dysfunction caused by synaptic vesicle circulation is strongly related to neurodegenerative diseases. Since the pathological changes of synapses generally occur in the early stage of neurodegenerative diseases [49,50], we reasonably speculate that patients may have synaptic dysfunction in the cerebral cortex after SARS-CoV-2 infection, including a loss of synapses and an inhibition of synaptic vesicle transport.

We identified two key modules and five hub genes based on the topological measures of the PPI network analysis. The GO pathway analysis of the dominant module was consistent with our previous results, which also highlighted that the synaptic vesicle cycle was the potential pathogenesis shared by COVID-19 and neurodegenerative diseases. Five hub genes, including *GAD2*, *SST*, *TAGLN3*, *SYP*, and *KCNJ4*, were further verified using the test_datasets of COVID-19, AD, and PD (Figure 6E).

*GAD2* is expressed in both pancreatic islets and the brain at later ages [51], particularly in the hypothalamus. It encodes one of the isoforms of glutamic acid decarboxylase enzyme, which is responsible for catalyzing the production of γ-aminobutyric acid (GABA) neurotransmitter. GABA is the primary inhibitory neurotransmitter in the central nervous system, and dysfunction of GABAergic mechanisms is associated with different neurological conditions. Previous studies have shown that stimulation of GABAergic signaling protects neurons against the neurotoxicity of amyloid β-protein. Therefore, *GAD2* might be a potential therapeutic target for AD treatment [52,53]. Somatostatin (SST), encoded by *SST,* is a well-known neuropeptide that is expressed throughout the brain. In the cortex, *SST* is expressed in a subset of GABAergic neurons and is known as a protein marker of inhibitory interneurons. Recent studies have identified the critical functions of *SST* in modulating cortical circuits in the brain and cognitive functions [54]. Furthermore, reduced expression of *SST* is a hallmark of various neurodegenerative and neuropsychiatric disorders, such as AD [55], PD [56], HD [57], major depressive disorder (MDD) [58], bipolar disorder, and schizophrenia (SCZ) [59]. *TAGLN3*, which is preferentially expressed in the CNS, is homologic to transgelin and calponin, two cytoskeleton-interacting proteins. *TAGLN3* is a member of the calponin family and co-localizes with actin and tubulin, which indicates that *TAGLN3* has a part in neuronal plasticity. Recently, Laurie et al. confirmed that *TAGLN3* was significantly down-regulated in the brain of patients with AD, and they considered it to be a molecular target to modulate neuroinflammation and a potential biomarker for AD [60]. SYP is a synaptic vesicle membrane protein that accounts for approximately 7–10% of the total vesicle proteins [61], and it is also used as a marker for synaptogenesis and synaptic density [62]. It has been reported that *SYP* could affect the efficiency of the synaptic vesicle cycle [63], which would then undermine cognitive ability. Schmitt et al. found *SYP* knock-out mice showed a significant dysfunction in learning and memory compared to wild-type mice, confirming the role of *SYP* in modulating cognitive functions [64]. *KCNJ4* encodes potassium voltage-gated channel subfamily J member 4, which is an inward rectifier potassium channel family member. Previous studies have shown that *KCNJ4* is associated with the progression and poor prognosis of lung adenocarcinoma [65], dilated cardiomyopathy [66], and prostate cancer [67]. Recent bioinformatic research revealed the ion channel-related gene features in COVID-19, of which the up-regulated gene, *KCNJ4*, was identified as the hub gene. This study indicated a correlation between *KCNJ4* and SARS-CoV-2 infection [68]. Moreover, Wang et al. showed that an overexpression of *KCNJ4* can protect against rotenone-induced apoptosis in cell models during the neurodegenerative process, suggesting the protective effect of *KCNJ4* on neurodegeneration [69].

Since the beginning of the pandemic, extensive global research studies have been underway to find appropriate drug agents to treat COVID-19. However, most of these drugs and therapies aim to reduce COVID-19-related hospitalization rates and deaths, without considering the improvement of neurological complications and ‘Long COVID’ [70,71]. Current treatments for post-COVID conditions are based on symptom relief and rehabilitation as there is no documented specific medical treatment [72]. Therefore, there is an urgent need for drugs to treat neurological symptoms related to COVID-19. Since developing a novel drug is a lengthy, expensive, and risky process, drug repurposing is the best approach to identify therapeutic options for COVID-19-related neurodegenerative diseases in a limited time [73]. Here, we identified candidate drugs from the DrugBank database, which contains very comprehensive information about approved drugs. Ibutilide fumarate is the first ‘pure’ class III intravenous antiarrhythmic agent indicated for the acute termination of atrial fibrillation and flutter [74]. Its predominant action is prolongation of the myocardial action potential duration through a unique ionic mechanism of action [75]. Dotarizine, a novel piperazine derivative, belongs to wide-spectrum Ca^2+^ channel antagonists. Compared to other Ca^2+^ channel blockers, Dotarizine was found to have a lower oral toxicity [76]. To date, there are no reports of these two drugs in the treatment of neurological diseases or COVID-19. They still need further experiments to explore their therapeutic potential in neurology. Azelnidipine, a long-acting calcium channel blocker, is highly lipid soluble and selective for the vascular wall [26]. Clinical studies have demonstrated that Azelnidipine markedly reduces heart rate and proteinuria in hypertensive patients by inhibiting sympathetic nerve activity. Azelnidipine has also been confirmed to have cardio-protective, neuroprotective, and anti-atherosclerotic properties, and it has also been found to prevent insulin resistance [77]. Many studies have reported its neuroprotective effects in ischemic stroke (IS) [26,78,79]. Since IS is considered an important contributing factor for the development of vascular dementia (VaD) and AD [80], Azelnidipine may also have neuroprotective effects on neurodegenerative diseases. Copper, a trace element, is present throughout the brain and is most prominent in the basal ganglia, hippocampus, cerebellum, numerous synaptic membranes, and in the cell bodies of cortical pyramidal and cerebellar granular neurons [81]. As a coenzyme factor, copper plays an important role in central nervous system development, and copper deficiency may result in neurological disorders [82,83,84]. Previous studies have found that copper is implicated directly or indirectly in the pathogenesis of numerous neurodegenerative diseases, such as AD, PD, ALS, and HD [83]. Artenimol is an artemisinin derivative and an antimalarial agent used in the treatment of uncomplicated *Plasmodium falciparum* infections. Recent evidence has demonstrated the potential effect of artemisinin against SARS-CoV-2 [85]. Nair et al. found that artemisia annua L. extracts inhibited the in vitro replication of SARS-CoV-2 and two of its variants [86]. Ruiz-Nuño et al. revealed that artemisinin and its derivatives portrayed more potent binding to Lys353 and Lys31-binding hotspots of SARS-CoV-2 spike protein than hydroxychloroquine, suggesting the potential repurposing of Artenimol for the treatment of COVID-19 [87].

## 4. Materials and Methods

### 4.1. Datasets Acquired in This Study

The transcriptome profiling used in this study was obtained from the GEO database (http://www.ncbi.nlm.nih.gov/geo/ accessed on 20 May 2022). The inclusion criteria of GSE188847 included the following: (a) severe COVID-19 patients with pre- or peri-mortem positive test results for SARS-CoV-2 by nasopharyngeal swab qPCR and history of hospitalization, and (b) age- (±2 years) and sex-matched uninfected controls without a history of neurological disorders or psychiatric diseases. The inclusion criteria of GSE150696 included the following: (a) all AD patients were diagnosed according to the Consortium to Establish a Registry for Alzheimer’s disease (CERAD); (b) all PD patients were selected on the basis of the Movement Disorders Society criteria; and (c) age- and sex-matched healthy people without a history of COVID-19.

In this study, one RNA-seq dataset of COVID-19 (GSE18847) and one array dataset containing AD and PD (GSE150696) patients were acquired as the training sets. To subsequently validate hub genes, we downloaded the GSE164332 dataset as a validation set for COVID-19, the GSE104704 dataset for AD, and two datasets, GSE20168 and GSE8397, for PD, which were merged, and their batch effects were corrected. Table 7 summarizes the detailed information of included datasets in this study.

### 4.2. Identification of Common DEGs among COVID-19, AD, and PD

Firstly, the “ComBat” function in the SVA package (version: 3.38.0) was applied to the merged datasets to correct batch effects. Next, we normalized the datasets and adjusted for covariates using the “Normalizebetweenarrays” and “removeBatchEffect” function in the limma package (version: 3.46.0) [88]. Principal component analysis (PCA), a classic dimension reduction approach, was conducted to verify intra-group data repeatability in each group using the FactoMineR package. A DEG is characterized as being expressed differently at the transcription level when there is a statistically significant difference between diverse conditions [89]. Herein, we performed differential expression analysis using the limma package to identify DEGs in R programming language (version: 4.1.3). The cutoff criteria (*p*-value < 0.05 and |logFC (fold change)| > 1) were applied to screen significant DEGs for all datasets. A Venn diagram analysis was performed to determine the shared and unique DEGs among COVID-19, AD, and PD.

### 4.3. Functional Annotation and Pathway Enrichment of Common DEGs

The Gene Ontology (GO) database provides a comprehensive and computational source to annotate gene product-based functions, comprising classes for molecular functions, the biological processes these contribute to, and the cellular locations where these occur [90]. Typically, the canonical pathway databases, Kyoto Encyclopedia of Genes and Genomes (KEGG) and Reactome, are considered as they are well-known databases to grasp the signaling and metabolic pathways [91]. Gene set enrichment analysis (GSEA) is another powerful analytical method for interpreting gene expression data [92]. The Disease Ontology (DO), a comprehensive and standardized knowledge base for inherited, developmental, and acquired human diseases, is utilized for disease annotation by major biomedical databases (e.g., Array Express, NIF, and IEDB) [93].

In this study, we performed enrichment analysis of GO, KEGG pathway, and GSEA for the common DEGs utilizing the clusterprofiler package (version: 3.18.1). The ReactomePA package was applied to the Reactome pathway analysis, and the DOSE package was employed for the Disease Ontology (DO) analysis. A *p*-value < 0.05 was considered statistically significantly different.

### 4.4. Construction of PPI Network and Key Module Analysis

The Search Tool for the Retrieval of Interacting Genes (STRING) (https://string-db.org/ accessed on 1 July 2022), which supplies experimental and predicted interaction-based information [94], was used to predict potential interactions between the identified common DEGs at the protein level with a medium confidence score. Additionally, Cytoscape software (version: 3.9) was used to construct and visualize the PPI network. Then, we used an important plugin of Cytoscape, Molecular Complex Detection (MCODE), to extract profound functional modules of genes in the PPI network with default parameters (K-core = 2, degree cutoff = 2, max. depth = 100, and node score cutoff = 0.2) [95]. The MCODE method is generally used to find densely connected regions in a PPI network that may represent molecular complexes or parts of pathways based on graph-theoretic clustering algorithms.

### 4.5. Detection of Hub Genes

Hub genes are identified as having high intramodular connectivity (or module membership), and previous research has revealed critical biological functions by assessing hub genes. To extract hub genes from the PPI network, we applied CytoHubba, which is a plugin of Cytoscape, to identify essential nodes and sub-networks from the complex interactome. It provides several topological algorithms that researchers can select (e.g., MCC, Degree, DMNC, MNC, EPC, and Bottleneck).

### 4.6. Identification of Candidate Drugs

The study of drug–target interaction is of great importance for drug discovery and design. Based on the common DEGs, candidate drugs and drug–target interactions were predicted using the DrugBank database (https://go.drugbank.com/ accessed on 10 July 2022), which is the world’s most widely used reference drug resource comprising detailed drug, drug–target, drug action, and drug interaction information about FDA-approved drugs, as well as experimental drugs going through the FDA approval process [96]. The intersection of the common DEGs and drug target genes (DTGs) downloaded from DrugBank was then used to identify related drugs. Finally, we excluded drugs that have an opposite effect on their target genes and acquired candidate drugs that might contribute to phenotypes. The statistical significance was set at *p*-value < 0.05.

### 4.7. Prediction of Transcription Factors

Precise regulation of gene expression is imperative for all biological processes. In this study, to identify substantial changes happening at the transcriptional level and obtain insights into the hub proteins’ regulatory molecules, we employed the RcisTarget package [97,98] to decode the regulatory transcription factors (TFs), and a *p*-value < 0.05 was considered significant. RcisTarget is an R-package to identify TF-binding motifs that are over-represented on a gene list.

## 5. Conclusions

Previous studies have shown that COVID-19 survivors are at high risk of neurodegenerative diseases [6], and degeneration of brain regions related to cognitive functions has been detected in milder cases [8]. It has also become evident that SARS-CoV-2 infection has a negative effect on the outcome of patients with neurodegenerative diseases. In the future, with an increasing number of infections, it is imperative to prevent or treat these neurological symptoms. Our study explored the relations among these three diseases in the context of transcriptomic analysis on AD, PD, and COVID-19 using bioinformatic analyses. We identified the five most significant hub genes from the common DEGs of these three diseases, and other transcriptome data can validate them. Most importantly, we found that the synaptic vesicle cycle was the common pathway shared by COVID-19, AD, and PD. Further analysis indicated that SARS-CoV-2 infection might lead to synaptic dysfunction and extensive synaptic down-regulation in the cortex of patients, thus triggering or aggravating neurodegenerative diseases. Our research contributes to a deeper understanding of the linkage of SARS-CoV-2 to neurodegenerative diseases, and it proposes potential therapeutic targets and related drugs, which may be promising therapeutic strategies for further clinical research studies.

Our study also had some limitations. Firstly, this research was performed based on bioinformatic and transcriptomic analyses; the differences in microarray platforms, tissue collection, RNA extraction methods, and statistical methods could produce potential bias in the results. In addition, our study was limited by the amount of available transcriptome expression data derived from the frontal cortex; thus, the size of the datasets used in this study needs to be larger to generate more compelling results. The inclusion of more large cohorts of COVID-19, AD, and PD patients should be better, and future cellular or animal experiments can also be conducted to provide convincing evidence to support our results. Therefore, the above findings should be taken with caution. Nevertheless, our study sheds light on the shared pathogenesis and molecular mechanism behind COVID-19, AD, and PD. Our results suggest the critical role of synaptic signaling and provide several promising genes for the potential correlation between SARS-CoV-2 infection and neurodegenerative diseases.

## Figures and Tables

**Table 1:** Clinical characteristics of the COVID-19_Train dataset.

	Total Sample, N (%)	COVID-19, N = 12 (50%), N (%)	Control, N = 12 (50%), N (%)	Statistics/*df*	*p*-Value
Gender (% female)	10 (41.67%)	5 (41.67%)	5 (41.67%)	0/1	1.0000
Age, in years, mean ± SD	66.79 ± 10.06	66.7 ± 10.38	66.9 ± 9.72	−0.0487/22	0.9616

**Table 2:** Clinical characteristics of the AD_Train dataset.

	Total Sample, N (%)	AD, N = 9 (50%), N (%)	Control, N = 9 (50%), N (%)	Statistics/*df*	*p*-Value
Gender (% female)	10 (55.56%)	5 (55.56%)	5 (55.56%)	0/1	1.0000
Age, in years, mean ± SD	85.17 ± 6.15	85.67 ± 6.36	84.67 ± 5.89	0.3461/16	0.7338
Postmortem interval, in hours, mean ± SD	44.11 ± 25.89	38.22 ± 23.30	50.0 ± 26.99	−0.9911/16	0.3364

**Table 3:** Clinical characteristics of the PD_Train dataset.

	Total Sample, N (%)	PD, N = 12 (57%), N (%)	PD, N = 9 (43%), N (%)	Statistics/*df*	*p*-Value
Gender (% female)	11 (52.4%)	6 (50%)	5 (55.56%)	0.0636/1	0.8008
Age, in years, mean ± SD	80.9 ± 6.78	78.08 ± 5.99	84.67 ± 5.89	−2.5125/19	0.0212
Postmortem interval, in hours, mean ± SD	40.48 ± 21.16	33.33 ± 10.87	50.0 ± 26.99	−1.9519/19	0.0659

**Table 4:** GO analysis results of common DEGs. The top ten enriched GO terms of each category are tabulated.

Category	GO ID	Term	*p*-Value	Gene ID
BP	GO:0006836	neurotransmitter transport	1.64 × 10^−5^	SYT5/SLC6A17/RAB3B/SYP/SLC17A7/SLC6A7
	GO:0006887	exocytosis	3.41 × 10^−5^	SYT5/PAK1/RAB3B/SYP/CRHBP/NSF/SYT13
	GO:0034765	regulation of ion transmembrane transport	3.70 × 10^−5^	KCNJ4/CACNG3/CACNB1/THY1/CRHBP/RASGRF1/KCNC2/CACNA1A
	GO:0051937	catecholamine transport	3.95 × 10^−5^	SYT5/RAB3B/SYT13/VIP
	GO:0015844	monoamine transport	6.85 × 10^−5^	SYT5/RAB3B/SYT13/VIP
	GO:0051952	regulation of amine transport	9.36 × 10^−5^	SYT5/RAB3B/SYT13/VIP
	GO:2001257	regulation of cation channel activity	0.0001	CACNG3/CACNB1/CRHBP/RASGRF1/KCNC2
	GO:0015837	amine transport	0.0001	SYT5/RAB3B/SYT13/VIP
	GO:0050804	modulation of chemical synaptic transmission	0.0001	SRGN/CACNG3/SYP/ADCYAP1/RASGRF1/NTNG1/CACNA1A
	GO:0099177	regulation of trans-synaptic signaling	0.0001	SRGN/CACNG3/SYP/ADCYAP1/RASGRF1/NTNG1/CACNA1A
CC	GO:0070382	exocytic vesicle	3.31 × 10^−10^	SYT5/SLC6A17/RAB3B/SYP/SVOP/SLC17A7/WDR7/ GAD2/ATP6V1G2/SYT13
	GO:0030672	synaptic vesicle membrane	4.42 × 10^−10^	SYT5/SLC6A17/RAB3B/SYP/SVOP/SLC17A7/GAD2/ ATP6V1G2
	GO:0099501	exocytic vesicle membrane	4.42 × 10^−10^	SYT5/SLC6A17/RAB3B/SYP/SVOP/SLC17A7/GAD2/ ATP6V1G2
	GO:0008021	synaptic vesicle	3.34 × 10^−9^	SYT5/SLC6A17/RAB3B/SYP/SVOP/SLC17A7/WDR7/ GAD2/ATP6V1G2
	GO:0030133	transport vesicle	8.86 × 10^−9^	SYT5/NRSN1/SLC6A17/RAB3B/SYP/SVOP/SLC17A7/WDR7/GAD2/ATP6V1G2/SYT13
	GO:0030658	transport vesicle membrane	8.37 × 10^−8^	SYT5/SLC6A17/RAB3B/SYP/SVOP/SLC17A7/GAD2/ ATP6V1G2
	GO:0098563	intrinsic component of synaptic vesicle membrane	1.54 × 10^−7^	SLC6A17/RAB3B/SYP/SLC17A7/ATP6V1G2
	GO:0150034	distal axon	1.01 × 10^−6^	NRSN1/SYP/THY1/CRHBP/ADCYAP1/RASGRF1/ KCNC2/OLFM1
	GO:0030285	integral component of synaptic vesicle membrane	2.19 × 10^−6^	SLC6A17/SYP/SLC17A7/ATP6V1G2
	GO:0043204	perikaryon	3.79 × 10^−6^	SERPINI1/CRHBP/ADCYAP1/KCNC2/OLFM1/VIP
MF	GO:0035254	glutamate receptor binding	6.19 × 10^−6^	CACNG3/NECAB2/NSF/RASGRF1
	GO:0022843	voltage-gated cation channel activity	3.14 × 10^−5^	KCNJ4/CACNG3/CACNB1/KCNC2/CACNA1A
	GO:0046873	metal ion transmembrane transporter activity	0.0001	KCNJ4/CACNG3/CACNB1/SLC17A7/KCNC2/ SLC6A7/CACNA1A
	GO:0005244	voltage-gated ion channel activity	0.0002	KCNJ4/CACNG3/CACNB1/KCNC2/CACNA1A
	GO:0022832	voltage-gated channel activity	0.0002	KCNJ4/CACNG3/CACNB1/KCNC2/CACNA1A
	GO:0000149	SNARE binding	0.0002	SYT5/NSF/SYT13/CACNA1A
	GO:0005245	voltage-gated calcium channel activity	0.0002	CACNG3/CACNB1/CACNA1A
	GO:0008331	high voltage-gated calcium channel activity	0.0003	CACNB1/CACNA1A
	GO:0019905	syntaxin binding	0.0009	SYT5/NSF/CACNA1A
	GO:0030165	PDZ domain binding	0.0013	KCNJ4/CACNG3/NSF

**Table 5:** Pathway enrichment analysis of common DEGs. The top 10 enriched pathways of the KEGG and Reactome databases are listed.

Category	ID	Pathway	*p*-Value	Gene ID
KEGG	hsa04721	Synaptic vesicle cycle	2.06 × 10^−6^	NSF/SLC17A7/ATP6V1G2/SLC6A7/ CACNA1A
	hsa04010	MAPK signaling pathway	0.0011	PAK1/CACNG3/CACNB1/RASGRF1/ CACNA1A
	hsa04727	GABAergic synapse	0.0019	NSF/GAD2/CACNA1A
	hsa04024	cAMP signaling pathway	0.0031	PAK1/SST/ADCYAP1/VIP
	hsa05033	Nicotine addiction	0.0056	SLC17A7/CACNA1A
	hsa04723	Retrograde endocannabinoid signaling	0.0079	NDUFV2/SLC17A7/CACNA1A
	hsa04921	Oxytocin signaling pathway	0.0089	KCNJ4/CACNG3/CACNB1
	hsa05120	Epithelial cell signaling in Helicobacter pylori infection	0.0163	PAK1/ATP6V1G2
	hsa05412	Arrhythmogenic right ventricular cardiomyopathy	0.0196	CACNG3/CACNB1
	hsa04260	Cardiac muscle contraction	0.0246	CACNG3/CACNB1
Reactome	R-HSA-112315	Transmission across chemical synapses	8.21 × 10^−7^	KCNJ4/CACNG3/CACNB1/NSF/SLC17A7/ RASGRF1/GAD2/CACNA1A
	R-HSA-112316	Neuronal system	1.84 × 10^−6^	KCNJ4/CACNG3/CACNB1/NSF/SLC17A7/ RASGRF1/KCNC2/GAD2/CACNA1A
	R-HSA-112308	Presynaptic depolarization and calcium channel opening	0.0005	CACNB1/CACNA1A
	R-HSA-5682910	LGI-ADAM interactions	0.0007	CACNG3/LGI2
	R-HSA-8986944	Transcriptional regulation by MECP2	0.0008	FKBP5/SST/GAD2
	R-HSA-422356	Regulation of insulin secretion	0.0015	SYT5/KCNC2/CACNA1A
	R-HSA-373080	Class B/2 (secretin family receptors)	0.0027	CRHBP/ADCYAP1/VIP
	R-HSA-112314	Neurotransmitter receptors and postsynaptic signal transmission	0.0029	KCNJ4/CACNG3/NSF/RASGRF1
	R-HSA-399719	Trafficking of AMPA receptors	0.0037	CACNG3/NSF
	R-HSA-399721	Glutamate binding, activation of AMPA receptors, and synaptic plasticity	0.0037	CACNG3/NSF

**Table 6:** The detailed information of the candidate drugs.

Drug	Type	*p*-Value	Target	Expression	Structure/Formula	Indication
Ibutilide	activator	0.0058	*CACNB1*	Down	C20H36N2O3S 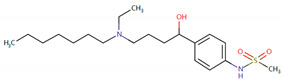	Indicated for the rapid conversion of atrial fibrillation or atrial flutter of recent onset to sinus rhythm.
Azelnidipine	agonist	0.0058	*CACNB1*	Down	C33H34N4O6 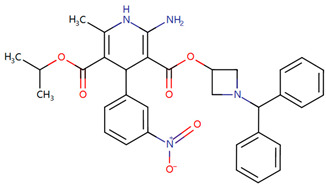	For the treatment of hypertension.
Dotarizine	unknow	0.0068	*CACNA1A*	Down	C29H34N2O2 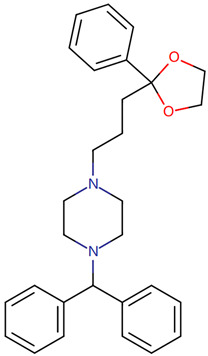	Investigated for use/treatment in migraine and cluster headaches.
Copper	unknow	0.0233	*SFPQ*	Up	Cu 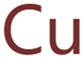	For use in the supplementation of total parenteral nutrition and in contraception with intrauterine devices.
Artenimol	ligand	0.0233	*SFPQ*	Up	C15H24O5 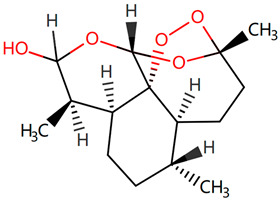	For the treatment of uncomplicated *Plasmodium falciparum* infection in adults, children, and infants aged 6 months and up and weighing over 5 kg. Used in combination with Piperaquine.

**Table 7:** Overview of datasets used in this study.

Disease	GEO Accession	Tissue Source	Data		DEG Count		GEO GPL	Assay Type
			Case	Control	Up	Down		
COVID-19 Train_dataset	GSE188847	frontal cortex	12	12	927	417	GPL24676	RNA-Seq
AD Train_dataset	GSE150696	frontal cortex	9	9	651	2004	GPL17585	Array
PD Train_dataset	GSE150696	frontal cortex	12	9	882	1707	GPL17585	Array
COVID-19 Val_dataset	GSE164332	frontal cortex	9	7	658	349	GPL18573	RNA-Seq
AD Val_dataset	GSE104704	lateral temporal lobe	12	18	683	2114	GPL18573	RNA-Seq
PD Val_dataset	GSE8397	frontal cortex	5	3	552	676	GPL96	Array
	GSE20168	frontal cortex	14	15	156	667	GPL96	Array

## Data Availability

The original contributions presented in the study are included in the article. Further inquiries can be directed to the corresponding author.

## Supplementary Materials

The following supporting information can be downloaded at https://www.mdpi.com/article/10.3390/ijms24054839/s1.
