# Supplementary material for: Exploration of the Shared Molecular Mechanisms between COVID-19 and Neurodegenerative Diseases through Bioinformatic Analysis
Source: Int J Mol Sci. 2023 Mar 2;24(5):4839. doi: 10.3390/ijms24054839 (PMC10002862; doi:10.3390/ijms24054839)
Supplement: Supplementary file 1 [file ijms-24-04839-s001.zip › ijms-2184897-supplementary.pdf]

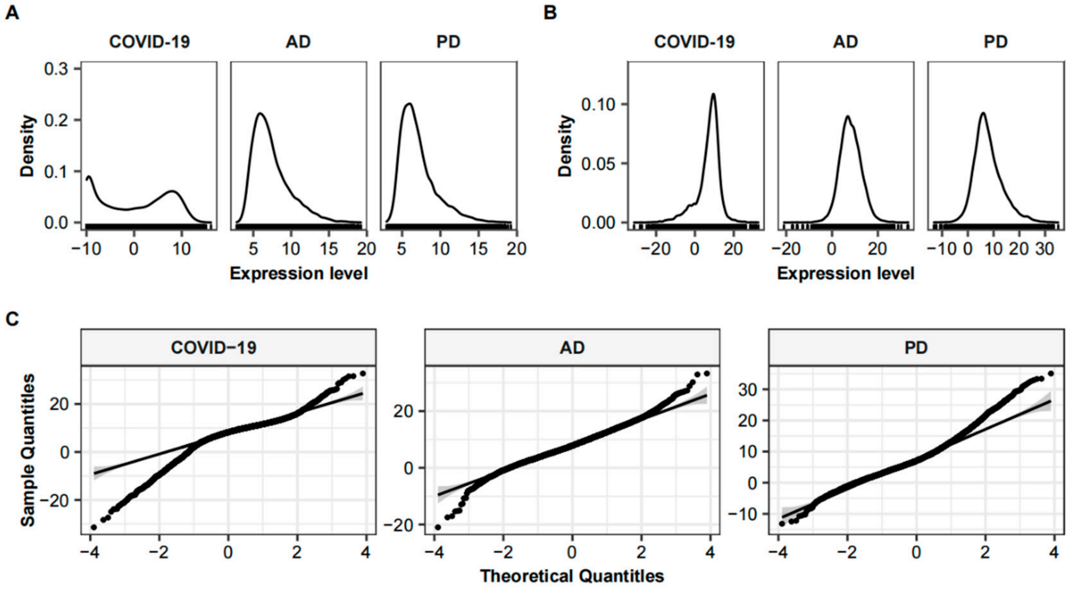

Supplementary Figure S1. The correction of COVID-19, AD, and PD train_datasets. The density plots of the train_datasets before (a) and after (b) normalization and removing batch-effects. (c) The Q-Q plots of the train_datasets after correction.

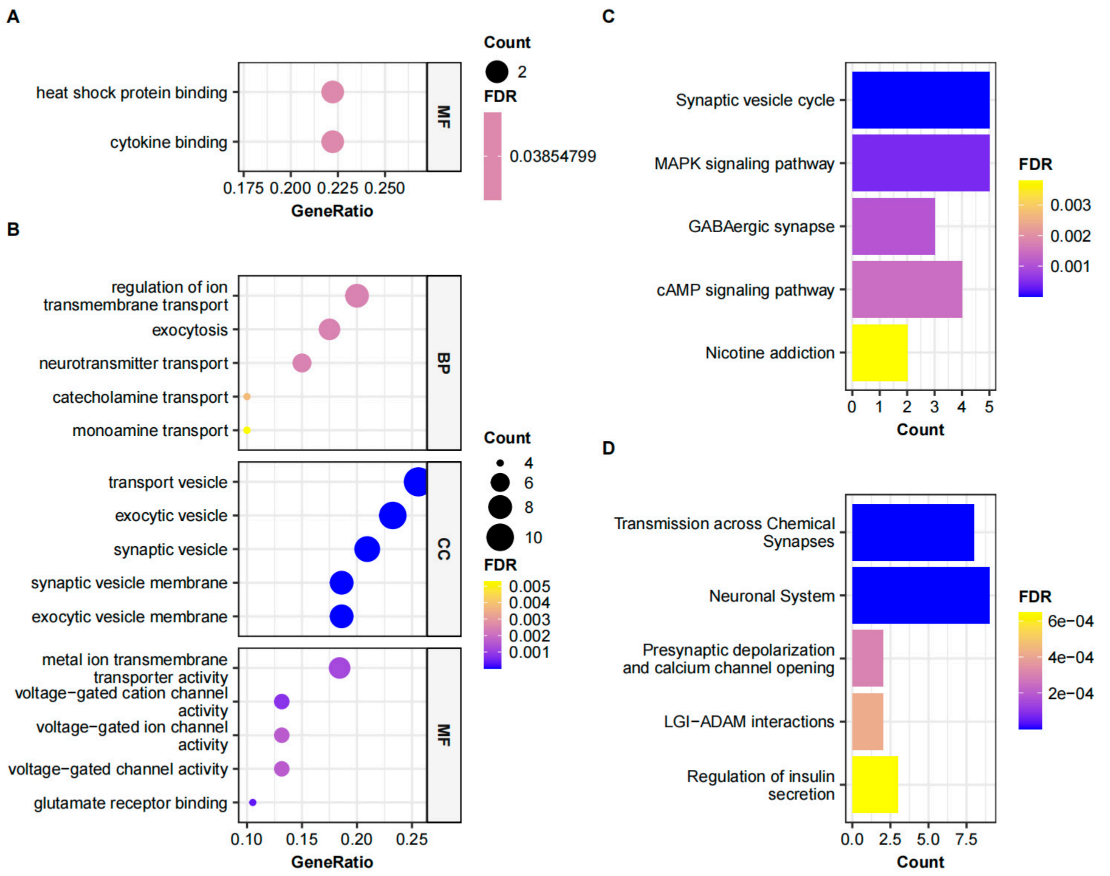

Supplementary Figure S2. Functional annotation of common up- and down-regulated DEGs. GO enrichment analysis of common up-regulated DEGs (a) and common down-regulated DEGs (b). (c) KEGG pathway enrichment of common down-regulated DEGs. (d) Reactome analysis of common down-regulated DEGs.
